# Efficacy of celastrol-supplemented diet in overweight and obese dogs: a 24-week randomized controlled trial

**DOI:** 10.3389/fvets.2025.1715988

**Published:** 2026-01-30

**Authors:** Sang-Yoon Shin, Woo-Jae Cho, Hun-Young Yoon, SooJung Lee, Jeong-Ik Lee, Mu-Young Kim

**Affiliations:** 1 Department of Veterinary Surgery, College of Veterinary Medicine, Konkuk University, Seoul, Republic of Korea; 2 Veterinary Nutrition Laboratory, Head of Research Center, Jeil Feed Company Ltd., Daejeon, Chungnam, Republic of Korea; 3 Laboratory of Biochemistry and Immunology, College of Veterinary Medicine, Chungbuk National University, Cheongju, Chungbuk, Republic of Korea; 4 KU Center for Animal Blood Medicine Science, Konkuk University, Seoul, Republic of Korea; 5 Regeniks Co., Ltd., Seoul, Republic of Korea; 6 Department of Companion Animal Health, Yeonsung University, Anyang, Gyeonggi, Republic of Korea; 7 Department of Veterinary Obstetrics and Theriogenology, College of Veterinary Medicine, Konkuk University, Seoul, Republic of Korea; 8 Department of Bio and Healing Convergence, Graduate School, Konkuk University, Seoul, Republic of Korea

**Keywords:** celastrol, companion animals, leptin, obesity, physical activity, weight management

## Abstract

Obesity is an increasingly common health issue among companion animals, contributing to various disorders and reduced life expectancy. Traditional management strategies such as caloric restriction and physical activity show limited long-term success, highlighting the need for alternative options. Celastrol, a leptin-sensitizing agent derived from Tripterygium wilfordii, has shown potent anti-obesity effects in rodent models; however, its effects in dogs have not been studied. This study evaluated the efficacy and safety of celastrol-supplemented feeding in overweight and obese dogs. Seventeen client-owned dogs (BCS ≥ 6) were randomly assigned to receive either a celastrol-supplemented diet or an isocaloric control diet for 24 weeks. Clinical parameters, including body weight, serum leptin concentrations, and physical activity were assessed at regular intervals. Dogs in the celastrol group exhibited a significantly greater reduction in body weight (mean 8.7%) compared to the control group (mean 2.1%) (*p* = 0.003). Serum leptin concentrations also decreased more markedly in the celastrol group, although no significant group-by-time interaction was detected. No significant differences were found between groups in thoracic and abdominal girth, BCS, MCS, physical activity, or blood chemistry. Importantly, no adverse clinical events were observed, and blood chemistry remained within reference ranges, supporting systemic safety. These findings suggest that celastrol supplementation effectively promotes weight loss without evident safety concerns in overweight and obese dogs. The results provide preliminary *in vivo* evidence for celastrol’s applicability in veterinary medicine and support its potential as an adjunctive strategy for obesity management. Further studies with larger and more diverse populations are warranted to validate these results.

## 1 Introduction

Obesity is an increasingly prevalent health issue among companion animals, affecting up to 40% in Western countries and reaching as high as 65% in specific populations. It arises from complex genetic, environmental, and behavioral factors and is strongly associated with diabetes mellitus, orthopedic disorders, respiratory dysfunction, and reduced life expectancy (1, 2).

Traditional weight management relies on dietary restriction and increased activity, typically using low-fat diets and exercise to enhance energy expenditure (3). However, their long-term effectiveness is often limited by poor owner compliance (4). Recently, pharmacological approaches such as appetite-modulating agents—including those targeting glucagon-like peptide-1 and peptide YY—have been introduced as alternatives (5, 6). Yet, these therapies often face receptor downregulation and desensitization, limiting long-term efficacy and requiring dose adjustments. This highlights the need for strategies that overcome behavioral and biological barriers for sustained weight control (6–8).

Celastrol, a pentacyclic triterpenoid extracted from *Tripterygium wilfordii*, has emerged as a promising anti-obesity agent primarily through its enhancement of leptin sensitivity and activation of the sympathetic nervous system, mechanisms that suppress appetite and increase thermogenesis (9, 10). In addition to these central effects, celastrol is also known to modulate energy metabolism through secondary pathways such as reduction of endoplasmic reticulum stress, and alterations in gut microbiota and bile acid profiles (10, 11). These multi-level actions position celastrol as a biologically versatile compound with potential applications in metabolic regulation for weight loss.

The anti-obesity effects of celastrol have been demonstrated in multiple *in vivo* studies, particularly in rodent obesity models, where celastrol produces pronounced reductions in body weight (9, 10, 12). Despite these findings in rodents, no studies to date have evaluated the *in vivo* effects of celastrol in companion animals. The aim of this study is to investigate the anti-obesity effects of celastrol-supplemented feed in dogs over a 24-week period by randomly assigning subjects to either a celastrol-fed or control group and monitoring changes in metabolic parameters. To the best of the authors’ knowledge, this is the first *in vivo* study to investigate the effects of celastrol in a canine model. By addressing this gap, the present research provides foundational data that may support the development of novel therapeutic strategies for obesity management in companion animals.

## 2 Materials and methods

### 2.1 Ethical approval

All procedures conducted in this study were approved by the Institutional Animal Care and Use Committee of the National Institute of Animal Science, Republic of Korea, and conducted in accordance with ethical standards for feed provision, physical examination, and follow-up assessments.

### 2.2 Subject recruitment and inclusion criteria

Client-owned neutered dogs aged 12 months or older were recruited at the Animal Hospital of Konkuk University. Three licensed veterinarians performed individual health assessments, including medical history interviews, physical examinations, and blood and urine analyses. The inclusion criteria were as follows: (1) No recent medications affecting metabolism or endocrine function (glucocorticoids, anticonvulsants, antihypertensives, antidiabetics) within 2 weeks. (2) Body Condition Score (BCS) ≥ 6 confirmed by all three evaluators using the 9-point scale; BCS 6–7 classified as overweight, BCS 8–9 as obese. (3) Endocrine screening: urinary cortisol-to-creatinine ratio <40 × 10^−6^, total T4 1.5–4.3 μg/dL, free T4 10–45 pmol/L, canine TSH < 0.5 ng/mL (12, 13). (4) Absence of inflammatory conditions; dogs with leukocytosis or elevated serum C-reactive protein were excluded (14).

### 2.3 Study design and dietary intervention

Dogs meeting inclusion criteria were enrolled for a total of 24 weeks between April and September 2019 at the Veterinary Teaching Hospital of Konkuk University. The study protocol and use of clinical data were approved by the Clinical Trial Center of Konkuk University. Participants were categorized as overweight (BCS 6–7) or obese (BCS 8–9) and then randomly assigned to the test (T) or control (C) group, with each BCS category evenly distributed between groups.

The T group received a celastrol-supplemented diet (Velixer O/F, Jeilfeed Inc., Daejeon, Korea), whereas the C group received an isocaloric diet identical in formulation except for the absence of celastrol. Daily feed allocation for both groups was calculated as 1.6 × resting energy requirement (RER = 70 × body weight^0.75^) and provided in two divided meals (15). Any uneaten food was discarded before the next feeding, and no additional food or treats were permitted. Celastrol used in the diet was derived from *Tripterygium wilfordii* root material supplied by the manufacturer. The extract was produced through an ethyl acetate–based extraction and purification process, and the celastrol content of the final extract was verified by high-performance liquid chromatography (HPLC). HPLC analysis confirmed a celastrol concentration of 0.743 mg/g in the extract. The test diet was formulated by incorporating the celastrol extract at a 2% inclusion rate. Based on the metabolizable energy of the diet (2972.5 kcal/kg) and the prescribed daily energy allowance, the estimated daily celastrol exposure in the test group averaged 0.35 mg/kg/day (range 0.28–0.41 mg/kg/day), depending on individual baseline body weight.

### 2.4 Clinical assessments

Physiological parameters were assessed at regular intervals during 24-week study. Body weight, thoracic girth, and abdominal girth were measured biweekly, and serum leptin concentrations at every 4 weeks. To account for individual size and breed variability, all measurements were normalized to baseline and expressed as percent change from week 0. BCS and Muscle Condition Score (MCS) were assessed at week 0 and 24. Potential adverse effects related to the celastrol-supplemented diet reported in previous literature were monitored through owner interviews and veterinarian observations, focusing on gastrointestinal disturbances (e.g., diarrhea) or respiratory distress (e.g., dyspnea) (16, 17). Systemic safety was assessed through complete blood count and blood chemistry (ALT, AST, BUN, creatinine, pro-BNP, CRP), measured at weeks 0 and 24 (18, 19).

### 2.5 Physical activity monitoring

All dogs were equipped with an PetPace accelerometer-based smart collar to evaluate physical activity by vital sign (20). The device monitored heart rate and movement in real time at two-week intervals, classifying physical activity into low, moderate, and high intensity level based on pulse reserve (PR), defined as the difference between a presumed maximum heart rate (200 bpm) and the individual’s resting heart rate. Low-intensity activity was defined as heart rates equal to the resting heart rate plus 50–60% of PR; moderate intensity as plus 61–80% of PR; high intensity as plus exceeding 80% of PR. The activity time at each intensity level and total activity time were recorded. The physical activity index (PAI) was calculated by assigning weights of 1, 2, and 3 to low, moderate, and high intensities, respectively, and summing the weighted results (21). Activity metrics were calculated over two-week intervals, using the value of the first 2 weeks as the reference baseline; Subsequent values were expressed as a percentage change from baseline value.

### 2.6 Statistical analysis

All statistical analyses were performed using GraphPad Prism version 8.02 (GraphPad Software Inc., San Diego, CA, United States). Independent *t*-tests were used to compare baseline clinical data between groups and to evaluate terminal between-group differences in body weight and serum leptin reduction. Longitudinal time-dependent trends in body weight, leptin, physical activity were analyzed with a linear mixed model (LMM). The model evaluated both within-group temporal changes and between-group differences in the rate of change. A *p*-value of <0.05 was considered statistically significant for all analyses.

## 3 Results

### 3.1 Study population

Total 18 client-owned dogs met the inclusion criteria and were enrolled in the study. 6 dogs were classified as obese (BCS 8–9) and 12 as overweight (BCS 6–7), with equal distribution of obese (3 each) overweight (6 each) category assigned to the C and T groups, resulting in 9 dogs per group. However, one overweight dog in the C group was lost for follow-up after week 4 and subsequently excluded from study. Therefore, the final composition consisted of 8 dogs in the C group (3 obese, 5 overweight) and 9 dogs in the T group (3 obese, 6 overweight).

### 3.2 Baseline characteristics of participants

**Figure 1:**
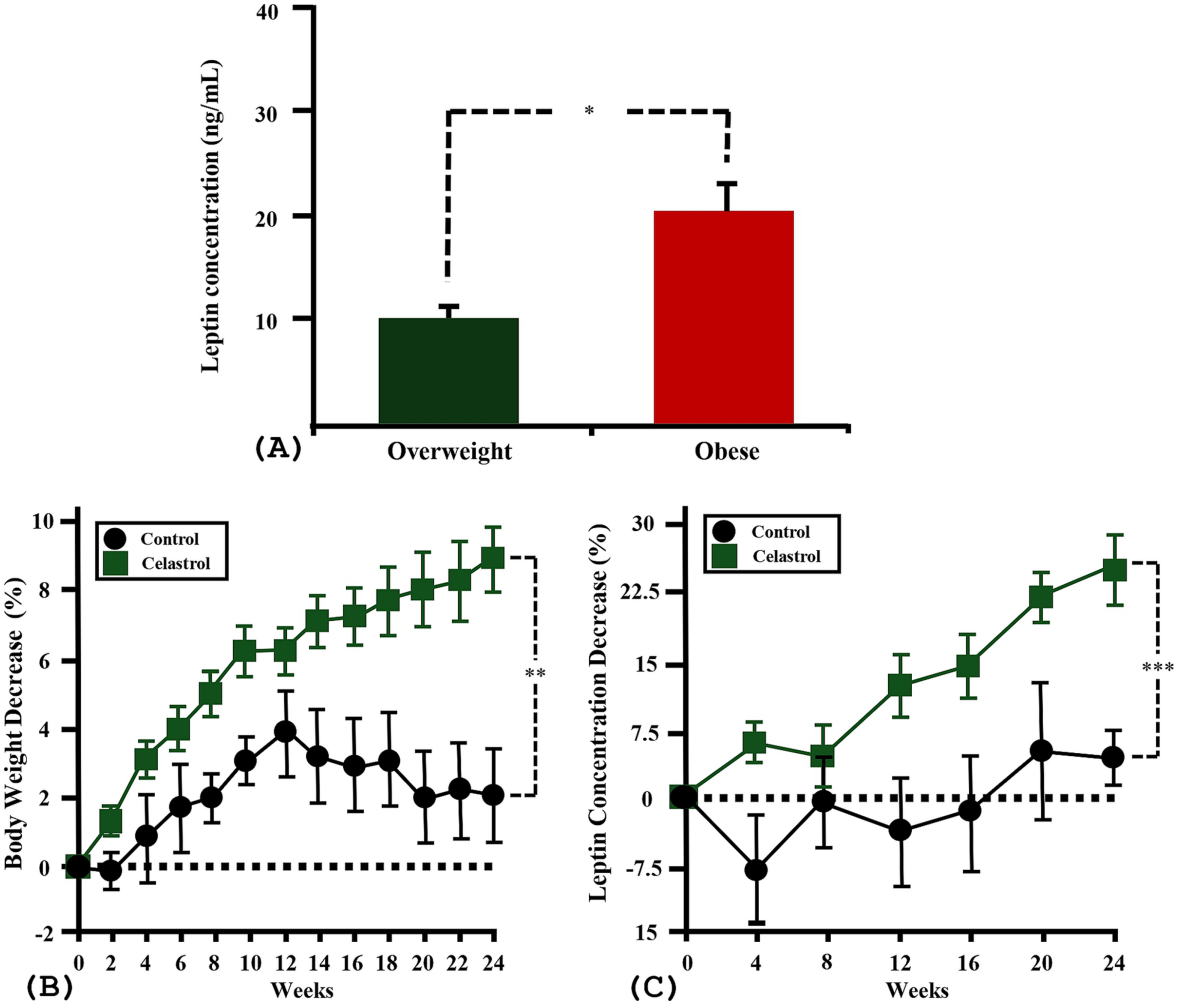
Baseline leptin differences by body condition and the effects of celastrol supplementation on body weight and serum leptin levels over time in overweight and obese dogs. **(A)** Baseline serum leptin concentrations (ng/mL) in overweight (*n* = 11) and obese (*n* = 6) dogs. Obese dogs exhibited significantly higher leptin levels compared to overweight dogs (*p* = 0.01). **(B)** Percent change in body weight from baseline to week 24 in control (C) and test (T) groups. The T group demonstrated significantly greater weight loss compared to the C group (*p* = 0.003), with a sustained downward trend over time (*p* < 0.001). **(C)** Percent change in serum leptin concentrations over 24 weeks. Leptin levels significantly declined in the T group (*p* < 0.001), but not in the C group (*p* = 0.1). No significant difference in time-dependent change patterns was observed between groups (*p* = 0.53). Error bars are represented as mean ± SEM. *p* values were determined by independent t-test and LMM (* *p* < 0.05, ** *p* < 0.01, *** *p* < 0.001).

The breed distribution in the C group included 4 Pomeranians, 2 Poodles, and 2 mixed breeds. The T group included 4 Pomeranians, 2 Poodles, and 1 each of Bichon Frisé, Cavalier King Charles Spaniel, and mixed breed. Regarding sex, the C group included 3 spayed females and 5 castrated males, while the T group included 5 spayed females and 4 castrated males. The average age was 5.3 ± 0.9 years (range 1–8 years) in the C group and 3.6 ± 0.9 years (range 1–10 years) in the T group, with no significant group difference (*p* = 0.22). The mean body weight was 5.7 ± 0.8 kg (range 2.7–9.6 kg) in the C group and 7.9 ± 1.4 kg (range 3.6–15.3 kg) in the T group, also showing no significant group difference (*p* = 0.21). The mean BCS was 5.9 ± 0.4 in the C group and 6.9 ± 0.3 in the T group, without a significant group difference (*p* = 0.98). Mean serum leptin concentration was 13.8 ng/mL (range 3.3–25.5 ng/mL) in the C group and 14.5 ng/mL (range 3.3–24.1 ng/mL) in the T group, with no significant group difference (*p* = 0.86) (Table 1). When comparing leptin concentrations by BCS classification, overweight dogs had significantly lower serum leptin levels (mean 10 ± 1.2 ng/mL, range 2.3–13.5 ng/mL) than obese dogs (mean 21.8 ± 3.2 ng/mL, range 9.2–31.6 ng/mL) (*p* = 0.01) (Figure 1A).

**Table 1:** Signalment of dogs in control and celastrol group (mean ± SE), [range].

Groups	Age (yrs.)	Body weight (kg)	BCS	Leptin (ng/mL)	Gender	Breed
Control group (*N* = 8)	5.3 ± 0.9 (1–8)	5.7 ± 0.8 (2.7–9.6)	6.9 ± 0.4 (6–9)	13.8 ± 2.8 (2.3–25.5)	Spayed female (3) Castrated male (5)	Pomeranian (4) Poodle (2) Mixed (2)
Test group (*N* = 9)	3.6 ± 0.9 (1–10)	7.9 ± 1.4 (3.6–15.2)	6.9 ± 0.3 (6–9)	14.5 ± 2.8 (3.3–24.1)	Spayed female (5) Castrated male (4)	Pomeranian (4) Poodle (2) Bichon (1) Cavalier King Charles Spaniel (1) Mixed (1)
*P*-value	0.22	0.21	0.98	0.86		

Breed, sex, age, body weight, Body Condition Score (BCS), and serum leptin concentrations at study entry. No statistically significant differences were observed between groups for any variable (*p* > 0.05). Values are presented as mean ± SEM, with ranges indicated in parentheses. Statistical comparisons were performed using independent *t*-tests for continuous variables and descriptive counts for categorical variables.

### 3.3 Effects on body weight

Comparison of body weight between baseline and week 24 showed a mean reduction of 2.1 ± 1.5% (range −4.1–6.6%) in the C group and 8.7 ± 1% (range 4.1–12.5%) in the T group, showing significant greater weight loss percentage of T group compared to the C group (*p* = 0.003). Time-based analysis of weight change trends revealed no significant pattern in the C group (*p* = 0.46), whereas the T group showed a consistent and statistically significant downward trend (*p* < 0.001). Comparison of the overall weight change trends confirmed that the T group experienced more sustained and significant weight reduction over time (*p* < 0.001) (Figure 1B).

### 3.4 Effects on serum leptin concentration

Serum leptin concentration also declined over the 24-week period, with the C group showing a mean reduction of 5 ± 4.4% (range −16.4–28.2%) and the T group showing a significantly greater mean reduction of 23 ± 2.5% (range 11.2–32.5%) (*p* = 0.003). Time-based analysis indicated that the C group did not exhibit a statistically significant decline in leptin levels (*p* = 0.12), while the T group showed a sustained and significant reduction throughout the study period (*p* < 0.001) (Figure 1). However, between-group comparison showed no statistically significant difference in leptin change trends (*p* = 0.53) (Figure 1C).

### 3.5 Effects on thoracic girth, abdominal girth, BCS, and MCS

**Figure 2:**
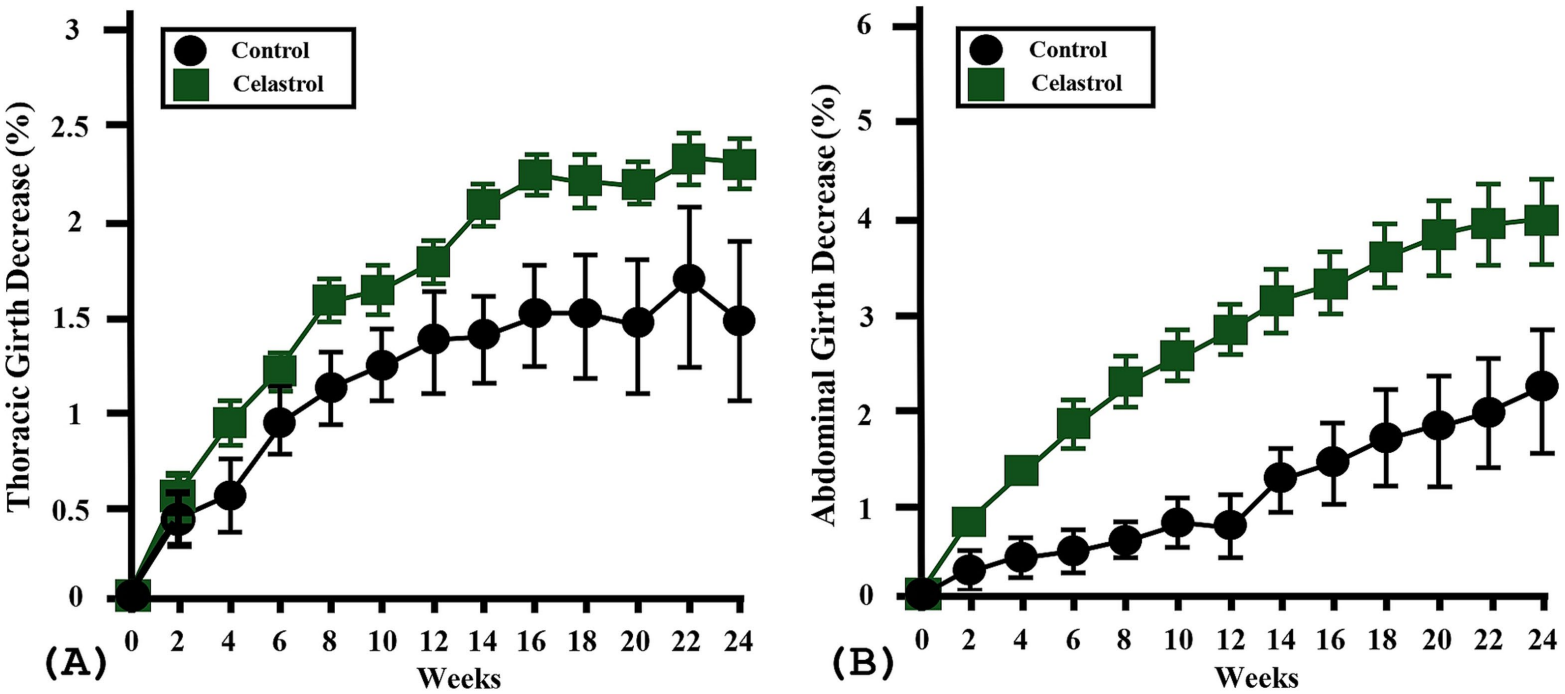
Effects of celastrol supplementation on thoracic and abdominal girth in overweight and obese dogs. **(A)** Percent change in thoracic girth from baseline to week 24 in control (C, *n* = 8) and test (T, *n* = 9) groups. Although the T group showed a slightly greater reduction (mean 2.3 ± 0.2%) compared to the C group (mean 1.5 ± 0.6%), the difference was not statistically significant (*p* = 0.23). **(B)** Percent change in abdominal girth from baseline to week 24 in control and test groups. Abdominal girth decreased more in the T group (mean 4 ± 0.5%) than in the C group (mean 2.2 ± 0.8%), but the group difference did not reach statistical significance (*p* = 0.09). Error bars are represented as mean ± SEM. *p* values were determined by independent *t*-test.

Thoracic girth decreased by 1.5 ± 0.6% (range −1.2–3.5%) in the C group and 2.3 ± 0.2% (range 1.9–3.2%) in the T group, with no statistically significant difference (*p* = 0.23). Abdominal girth showed a reduction of 2.2 ± 0.8% (range −3.1–4.3%) in the C group and 4 ± 0.5% (range 1.9–6.1%) in the T group, which also did not show significant difference (*p* = 0.09). The BCS decreased by 0 or 1 point in all participants; mean changes were 0.4 ± 0.2 in the C group and 0.7 ± 0.2 in the T group, with no significant group difference (*p* = 0.26) (Figure 2). No dogs showed MCS change between baseline and week 24. No respiratory or gastrointestinal symptoms were observed during the study period, and no dogs demonstrated clinically relevant elevations in serum ALT, AST, BUN, creatinine, pro-BNP, or CK at week 24 compared to baseline.

### 3.6 Effects on physical activity trends

**Figure 3:**
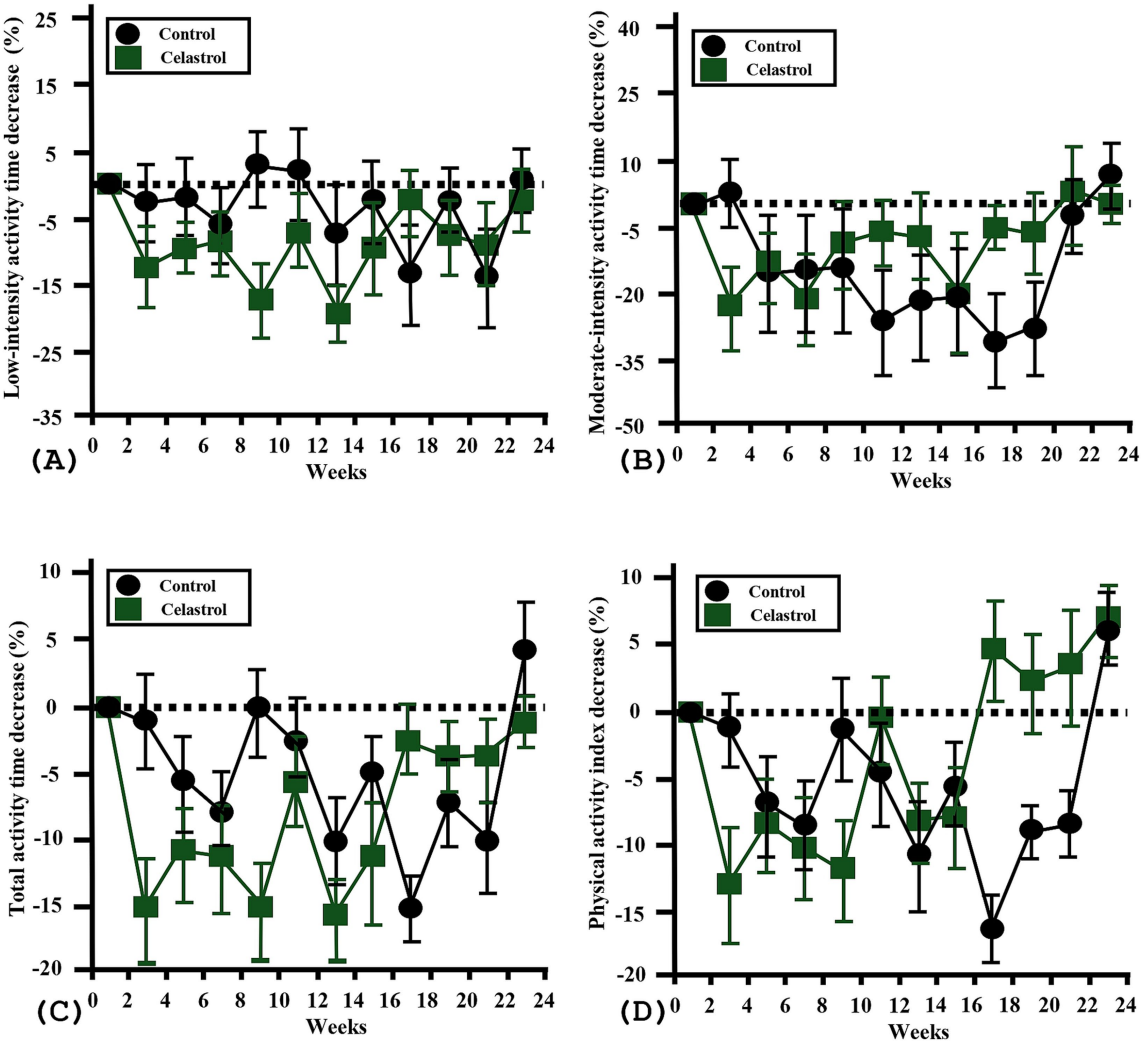
Longitudinal analysis of physical activity metrics in celastrol-supplemented versus control groups. **(A)** Low-intensity activity time across the 24-week period. No statistically significant changes were observed over time in either the control (*p* = 0.18) or test group (*p* = 0.26). The group-by-time interaction also showed no.17 significant difference (*p* = 0.08). **(B)** Moderate-intensity activity time over time. No significant time-dependent changes were observed in either group (control: *p* = 0.93; test: *p* = 0.74), and between-group trends did not differ significantly (*p* = 0.82). **(C)** Total activity time, regardless of intensity. No within-group significance was noted (control: *p* = 0.23; test: *p* = 0.27), nor was there a significant difference in overall trends between groups (*p* = 0.1). **(D)** Physical Activity Index (PAI), calculated based on weighted activity intensity. PAI remained stable over time in both groups (control: *p* = 0.36; test: *p* = 0.3), and no group-by-time interaction was found (*p* = 0.17). Error bars are shown as mean ± SEM. Time effects and interaction effects were analyzed using linear mixed models (LMM).

No high-intensity activity was recorded for any participant during the study period; therefore, physical activity was assessed using only low and moderate intensity metrics. Time-based trends in low intensity activity time, moderate intensity activity time, total activity time, and PAI were analyzed. In the C group, no statistically significant time-dependent changes were observed in any activity metric (low: *p* = 0.18; moderate: *p* = 0.93; total: *p* = 0.23; PAI: *p* = 0.36). Similarly, in the T group, none of the activity parameters showed significant changes over time (low: *p* = 0.26; moderate: *p* = 0.74; total: *p* = 0.27; PAI: p = 0.3). Group-by-time interactions also revealed no significant differences in the trend of activity changes between groups (low: *p* = 0.08; moderate: *p* = 0.82; total: *p* = 0.1; PAI: *p* = 0.17). These findings suggest that physical activity levels remained stable throughout the study in both groups, with no significant divergence between two groups (Figure 3).

## 4 Discussion

To date, celastrol-containing extracts have been evaluated for their anti-obesity effects only in *in vitro* canine adipocyte studies, with no prior *in vivo* investigations in dogs or cats (22). Oral celastrol has otherwise been examined mainly in rodent models, typically at high doses and over short durations. For example, celastrol administered at 12.5 mg/kg/day for several days in rats, or at 10 mg/kg/day for 3 weeks in diet-induced obese mice, resulted in rapid and marked weight loss but with an early plateau (10, 17). In contrast, our study delivered substantially lower dietary exposures over a prolonged 24-week period, yielding a modest but sustained reduction in body weight. Celastrol’s primary mechanism—restoration of leptin responsiveness by alleviating obesity-associated leptin resistance—may explain why continued efficacy was observed in our cohort, as no dog reached a lean condition and leptin levels likely remained sufficient to maintain a leptin-sensitizing response (10, 11, 16, 19). Longer-term studies and inclusion of lean dogs will be required to further characterize dose–response patterns and duration of effect.

Several rodent studies have reported dose-dependent toxicities of celastrol including gastrointestinal, respiratory, hepatic adverse effects mainly at oral doses ≥7.5 mg/kg/day (16–19). In our study, no clinically relevant adverse events, blood test abnormalities, or reductions in MCS were observed, which is consistent with the substantially lower exposure delivered through the diet (10). Although these doses are well below rodent toxicity thresholds, species-specific pharmacokinetic differences and celastrol’s lipophilicity raise the possibility of tissue accumulation (23, 24). Therefore, comprehensive long-term pharmacokinetic and toxicological studies are needed to define safe exposure ranges for dogs.

An average weight reduction of 2.1% was also observed in the control group. The high-protein and low-fat composition of the diet, together with strict exclusion of additional foods or treats, may have contributed to this modest loss (25–27). Omega-3 fatty acids and L-carnitine included in the diet could also have supported metabolic stability and fat oxidation (28–30). In addition, strict adherence to controlled feeding schedules and the possibility that the study diet was less palatable than the dogs’ usual diets may have reduced voluntary intake, contributing to weight loss independent of celastrol.

Although leptin levels in the celastrol group showed a more pronounced decline, the slope did not differ significantly from that of the control group. The sharper decrease in the T group is consistent with enhanced leptin sensitivity and reduced adiposity (10, 11, 16). However, group-by-time differences may have been attenuated by the multifactorial regulation of leptin, including individual variation in metabolic and hormonal status or low-grade inflammation undetected on routine blood tests, as well as natural reductions in body fat in both groups resulting from standardized RER-based feeding without treats (31–33). These factors likely reduced inter-group separation in leptin trajectories despite the greater absolute decrease observed in the celastrol group.

Physical activity levels remained stable in both groups, with no significant within- or between-group differences in activity time or PAI (21). This suggests that celastrol did not influence activity under the controlled-feeding conditions used in this study. Although mouse studies reported reduced locomotor activity by suppressed food-seeking behavior, such effects may be minimized in dogs receiving fixed scheduled meals (10). The PAI metric system provided an individualized measure of activity intensity but does not fully correspond to oxygen-consumption-based systems such as Metabolic Equivalent of Task used in humans (34). Accordingly, PAI-derived analyses should be interpreted with caution.

This study has several limitations inherent to its pilot-scale design. First, only overweight and obese dogs were included in study, so the effects of celastrol in lean individuals remain unknown. Because celastrol’s pharmacological actions may vary with adiposity, future trials should adopt broader body condition categories. Second, although diets were standardized based on calculated RER, actual food intake was not quantitatively recorded Given that celastrol’s anti-obesity effects are largely attributed to leptin sensitization and an anorexigenic response, reduced food consumption in the treated group may have contributed to weight loss. However, inconsistent owner reporting prevented reliable analysis and future studies should include objective dietary tracking of food intake. Third, the relatively small sample size raises the possibility of type II error. Voluntary recruitment also resulted in heterogeneity in breed and sex distribution, producing baseline imbalance in initial body weight and in the proportion of neutered females, which are predisposed to greater adipose accumulation (35, 36). Because neutered females were more represented in the treated group, this imbalance is unlikely to have contributed to the greater weight reduction observed. Moreover, weight change was evaluated as a percentage rather than an absolute reduction, which further limits the practical impact of baseline disparities. Nevertheless, larger studies with breed- and sex-matched cohorts will be necessary to provide more objective and generalizable evidence. Fourth, a dose of celastrol could not be standardized across dogs as the compound was incorporated into the diet rather than administered as a purified dose. RER-based feeding inherently led to variation in celastrol intake across individuals. Future investigations should evaluate fixed-dose administration to clarify dose–response relationships.

In summary, celastrol supplementation provided a distinct advantage over dietary restriction alone in promoting weight loss in overweight and obese dogs, without evidence of clinically relevant adverse effects. Although reductions in leptin were more pronounced in the celastrol group, inter-group differences in time-dependent trends were not significant, highlighting the complexity of leptin regulation. These findings provide the first *in vivo* evidence supporting celastrol as a promising adjunctive strategy for obesity management in dogs. Future investigations with larger, breed-controlled populations, inclusion of lean individuals, and detailed food intake monitoring are warranted to establish its long-term efficacy and safety.

## Data Availability

The raw data supporting the conclusions of this article will be made available by the authors, without undue reservation.
